# Quantum State Tomography via Linear Regression Estimation

**DOI:** 10.1038/srep03496

**Published:** 2013-12-13

**Authors:** Bo Qi, Zhibo Hou, Li Li, Daoyi Dong, Guoyong Xiang, Guangcan Guo

**Affiliations:** 1 Key Laboratory of Systems and Control, ISS, and National Center for Mathematics and Interdisciplinary Sciences, Academy of Mathematics and Systems Science, CAS, Beijing 100190, P. R. China; 2 Key Laboratory of Quantum Information, University of Science and Technology of China, CAS, Hefei 230026, P. R. China; 3 School of Engineering and Information Technology, University of New South Wales at the Australian Defence Force Academy, Canberra, ACT 2600, Australia

## Abstract

A simple yet efficient state reconstruction algorithm of linear regression estimation (LRE) is presented for quantum state tomography. In this method, quantum state reconstruction is converted into a parameter estimation problem of a linear regression model and the least-squares method is employed to estimate the unknown parameters. An asymptotic mean squared error (MSE) upper bound for all possible states to be estimated is given analytically, which depends explicitly upon the involved measurement bases. This analytical MSE upper bound can guide one to choose optimal measurement sets. The computational complexity of LRE is *O*(*d*^4^) where *d* is the dimension of the quantum state. Numerical examples show that LRE is much faster than maximum-likelihood estimation for quantum state tomography.

One of the essential tasks in quantum technology is to verify the integrity of a quantum state^1^. Quantum state tomography has become a standard technology for inferring the state of a quantum system through appropriate measurements and estimation^2^,^3^,^4^,^5^,^6^,^7^,^8^. To reconstruct a quantum state, one may first perform measurements on a collection of identically prepared copies of a quantum system (data collection) and then infer the quantum state from these measurement outcomes using appropriate estimation algorithms (data analysis). Measurement on a quantum system generally gives a probabilistic result and an individual measurement outcome only provides limited information on the state of the system, even when an ideal measurement device is used. In principle, an infinite number of measurements are required to determine a quantum state precisely. However, practical quantum state tomography consists of only finite measurements and appropriate estimation algorithms. Hence, the choice of optimal measurement sets and the design of efficient state reconstruction algorithms are two critical issues in quantum state tomography.

Many results have been presented for choosing optimal measurement sets to increase the estimation accuracy and efficiency in quantum state tomography^9^,^10^,^11^. Several sound choices that can provide excellent performance for tomography are, for instance, tetrahedron measurement bases, cube measurement sets, and mutually unbiased bases^11^. However, for most existing results, the optimality of a given measurement set is only verified through numerical results^11^. There are few methods that can analytically give an estimation error bound^12^,^13^,^14^, which is essential to evaluate the optimality of a measurement set^15^,^16^,^17^ and the appropriateness of an estimation method.

For estimation algorithms, several useful methods including maximum-likelihood estimation (MLE)^2^,^18^,^19^,^20^,^21^, Bayesian mean estimation (BME)^2^,^22^,^23^ and least-squares (LS) inversion^24^ have been proposed for quantum state reconstruction. The MLE method simply chooses the state estimate that gives the observed results with the highest probability. This method is asymptotically optimal in the sense that the estimation error can asymptotically achieve the Cramér-Rao bound. However, MLE usually involves solving a large number of nonlinear equations where their solutions are notoriously difficult to obtain and often not unique. Recently, an efficient method has been proposed for computing the maximum-likelihood quantum state from measurements with additive Gaussian noise, but this method is not general^21^. Compared to MLE, BME can always give a unique state estimate, since it constructs a state from an integral averaging over all possible quantum states with proper weights. The high computational complexity of this method significantly limits its application. The LS inversion method can be applied when measurable quantities exist that are linearly related to all density matrix elements of the quantum state being reconstructed^24^. However, the estimation result may be a nonphysical state and the mean squared error (MSE) bound of the estimate cannot be determined analytically.

Here, we present a new linear regression estimation (LRE) method for quantum state tomography that can identify optimal measurement sets and reconstruct a quantum state efficiently. We first convert the quantum state reconstruction into a parameter estimation problem of a linear regression model^25^. Next, we employ an LS algorithm to estimate the unknown parameters. The positivity of the reconstructed state can be guaranteed by an additional least-squares minimization problem. The total computational complexity is *O*(*d*^4^) where *d* is the dimension of the quantum state. In order to evaluate the performance of a chosen measurement set, an MSE upper bound for all possible states to be estimated is given analytically. This MSE upper bound depends explicitly upon the involved measurement bases, and can guide us to choose the optimal measurement set. The efficiency of the method is demonstrated by examples on qubit systems.

## Results

### Linear regression model

We first convert the quantum state tomography problem into a parameter estimation problem of a linear regression model. Suppose the dimension of the Hilbert space 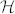 of the system of interest is *d*, and 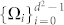 is a complete basis set of orthonormal operators on the corresponding Liouville space, namely, 
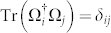
, where † denotes the Hermitian adjoint and *δ_ij_* is the Kronecker function. Without loss of generality, let 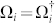 and 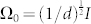, such that the other bases are traceless. That is Tr(Ω*_i_*) = 0, for 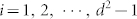. The quantum state *ρ* to be reconstructed may be parameterized as 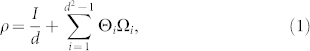
where Θ*_i_* = Tr(*ρ*Ω*_i_*). Given a set of measurement bases 
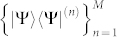
, each |Ψ〉〈Ψ|^(*n*)^ can be parameterized under the bases 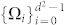 as 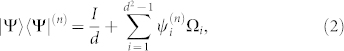
where 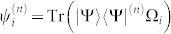.

When one performs measurements with measurement set 
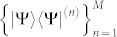
 on a collection of identically prepared copies of a quantum system (with state *ρ*), the probability to obtain the result of |Ψ〉〈Ψ|^(*n*)^ is 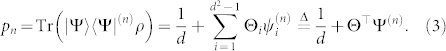
Assume that the total number of experiments is *N* and *N*/*M* experiments are performed on *N*/*M* identically prepared copies of a quantum system for each measurement basis |Ψ〉〈Ψ|^(*n*)^. Denote the corresponding outcomes as 
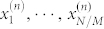
, which are independent and identically distributed. Let 
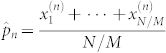
 and 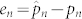. According to the central limit theorem^26^, *e_n_* converges in distribution to a normal distribution with mean 0 and variance 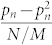. Using (3), we have the linear regression equations for 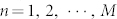, 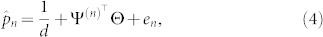
where ⊤ denotes the matrix transpose.

Note that 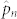, *d* and Ψ^(*n*)^ are all available, while *e_n_* may be considered as the observation noise whose variance is asymptotically 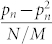. Hence, the problem of quantum state tomography is converted into the estimation of the unknown vector Θ. Denote 
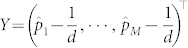
, 
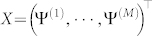
, 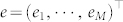. We can transform the linear regression equations (4) into a compact form 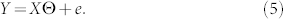
We define the MSE as 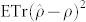, where 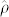 is an estimate of the quantum state *ρ* based on the measurement outcomes and E(**·**) denotes the expectation on all possible measurement outcomes. For a fixed tomography method, 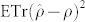 depends on the state *ρ* to be reconstructed and the chosen measurement bases. From a practical viewpoint, the optimality of a chosen set of measurement bases may rely upon prior information but should not depend on any specific unknown quantum state to be reconstructed. In this paper, no prior assumption is made on the state *ρ* to be reconstructed. Given a fixed tomography method, we use the maximum MSE for all possible states (i.e., 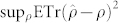) as the index to evaluate the performance of a chosen set of measurement bases.

### Linear regression estimation

To give an estimate with high level of accuracy and low computational complexity, we employ the LS method, where the basic idea is to find an estimate 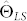 such that 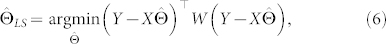
where 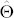 is an estimate of Θ, and *W* is a diagonal weighting matrix. Since the objective function is quadratic, one has the LS solution as follows: 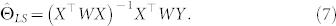
The LS solution (7) can be calculated in a recursive way (see the Methods section). In practical experiments, the cost of time can be greatly reduced by employing a recursive reconstruction protocol since the estimate can be calculated recursively based on available data at the same time of performing measurements to acquire data.

Note that if *p_n_* = 1, we have already reconstructed the state as |Ψ〉〈Ψ|^(*n*)^; if *p_n_* = 0, we should choose the following measurement basis from the orthogonal complementary space of |Ψ〉〈Ψ|^(*n*)^. Hence, in general the smaller the variance of *e_n_* is, the more the information can be extracted by |Ψ〉〈Ψ|^(*n*)^. Therefore, the corresponding weight of the *n*-th regression equation should be bigger. It can be verified that if all *p_n_* are known, the LS soution 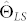 satisfying 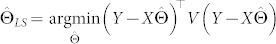 is asymptotically the minimum variance unbiased estimator of Θ, where *V* is the inverse of 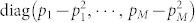. Hence, an appropriate choice of *W* is the inverse of 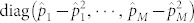.

However, for simplicity we consider the case where *W* = *I*, and the corresponding LS solution is 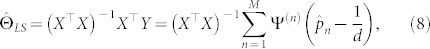
where 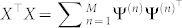.

If the measurement bases 
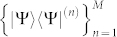
 are informationally complete or overcomplete, *X*^T^*X* is invertible. Using (5), (8) and the statistical property of the observation noise 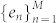 (independent and asymptotically Gaussian), the estimate 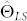 has the following properties for a fixed set of chosen measurement bases:

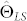
 is asymptotically unbiased;
2. The MSE 
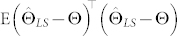
 of 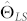 is asymptotically 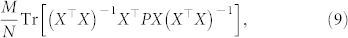
where 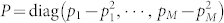.

Note that *p_n_* depends upon the state to be reconstructed and the measurement basis |Ψ〉〈Ψ|^(*n*)^ for 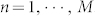. Recall that the optimality of a chosen set of measurement bases should not depend upon any specific unknown quantum state to be reconstructed. We can take the supremum of equation (9) under all possible states to get the performance index for any given set of measurement bases 
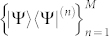
 as 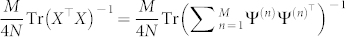.

### Positivity and computational complexity

Based on the solution 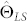 obtained from (8), we can obtain a Hermitian matrix 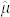 with 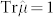 using (1). However, 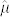 may have negative eigenvalues and be nonphysical due to the randomness of measurement results. In this sense, 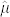 is called pseudo linear regression estimation (PLRE) of state *ρ*. A good method of pulling 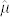 back to a physical state can reduce the MSE. In this paper, the physical estimate 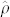 is chosen to be the closest density matrix to 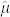 under the matrix 2-norm. In standard state reconstruction algorithms, this task is computationally intensive^21^. However, we can employ the fast algorithm in^21^ with computational complexity *O*(*d*^3^) to solve this problem since we have obtained a Hermitian estimate 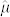 with 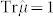.

**Figure 1:**
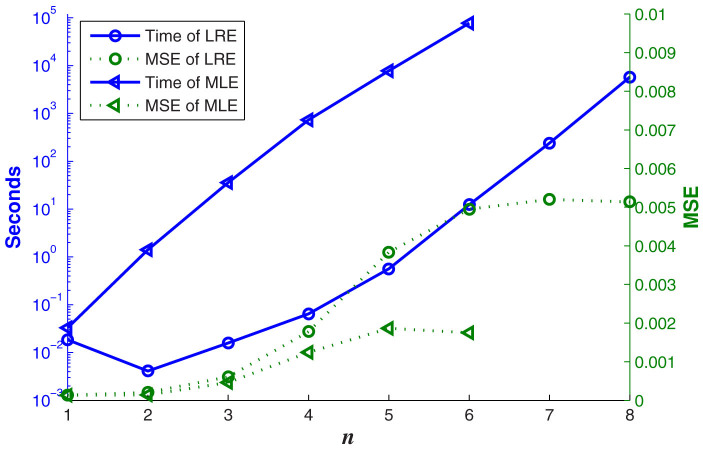
The run time and MSE of LRE and MLE for random *n*-qubit pure states mixed with the identity^21^. The realization of MLE used the iterative method in^2^. The measurement bases are from the *n*-qubit cube measurement set and the resource is *N* = 3^9^ × 4*^n^*. The simulated measurement results for every basis |Ψ〉〈Ψ|^(*i*)^ are generated from a binomial distribution with probability *p_i_* = Tr(|Ψ〉〈Ψ|^(*i*)^*ρ*) and trials *N*/*M*. LRE is much more efficient than MLE with a small amount of accuracy sacrificed since the maximum MSE could reach 2 for the worst estimate. All timings were performed in MATLAB on the computer with 4 cores of 3 GHz Intel i5-2320 CPUs.

Since an informationally complete measurement set 
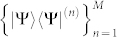
 requires *M* being *O*(*d*^2^), the computational complexity of (1) and *X*^T^*Y* in (8) is *O*(*d*^4^). Although the computational complexity of calculating (*X*^T^*X*)^−1^ is generally *O*(*d*^6^), (*X*^T^*X*)^−1^ can be computed off-line before the experiment once the measurement set is determined. Hence, the total computational complexity of LRE after the data have been collected is *O*(*d*^4^). It is worth pointing out that for *n*-qubit systems, 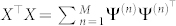 is diagonal for many preferred measurement sets such as tetrahedron and cube measurement sets. Fig. 1 compares the run time of our algorithm with that of a traditional MLE algorithm. Since the maximum MSE could reach 2 for the worst estimate, it is clear that our state reconstruction algorithm LRE is much more efficient than MLE with a small amount of accuracy sacrificed.

### Optimality of measurement bases

One of the advantages of LRE is that the MSE upper bound can be given analytically as 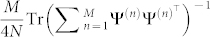, which is dependant explicitly upon the measurement bases. Note that if the PLRE 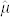 is a physical state, then the MSE upper bound is asymptotically tight for the evaluation of the performance of a fixed set of measurement bases. Hence, to choose an optimal set 
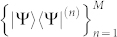
, one can solve the following optimization problem: 
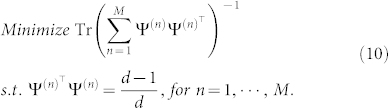
The optimization problem can be solved in an off-line way by employing appropriate algorithms though it may be computationally intensive. We will discuss this problem in other work.

With the help of the analytical MSE upper bound, we can ascertain which one is optimal among the available measurement sets. This is demonstrated when we prove the optimality of several typical sets of measurement bases for 2-qubit systems.

For 2-qubit systems, it is convenient to chose 
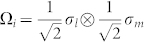
, where *i* = 4*l* + *m*; *l*, *m* = 0, 1, 2, 3; *σ*_0_ = *I*_2×2_, 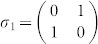, 
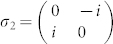
, 
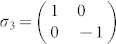
.

If the form of the measurement bases is not restricted, the minimum of the MSE upper bound 
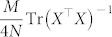
 for all possible measurement bases is 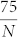. This minimum can be reached by using the mutually unbiased measurement bases. While as in many practical experiments, if only local measurements can be performed, the minimum of the MSE upper bound 
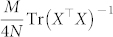
 is 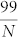. This minimum can be reached by using the 2-qubit cube or tetrahedron measurement set.

**Figure 2:**
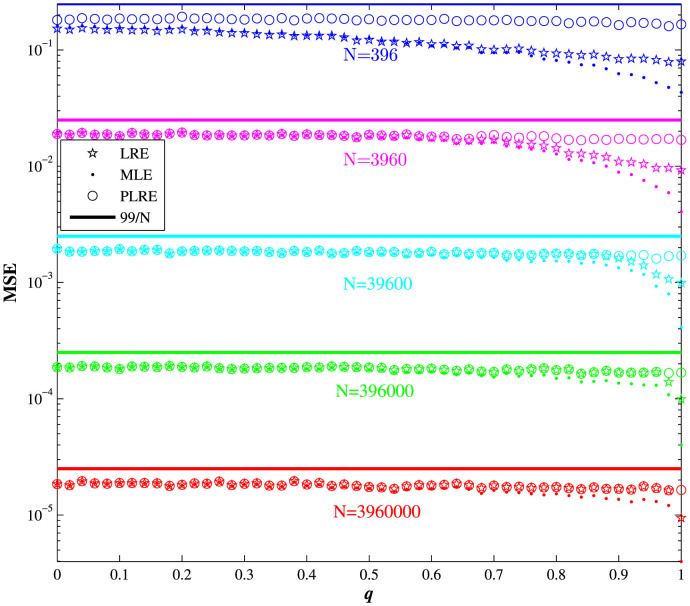
Mean squared error (MSE) for Werner states (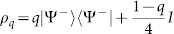 with 
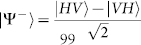
) with *q* (varying from 0 to 1) and different numbers of copies *N*. The cube measurement set is used, where the MSE upper bound is 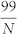. It can be seen that the MSE of PLRE is almost unchanged for *q* ∈ [0, 1], and is larger than the MSE of LRE.

Fig. 2 shows the dependant relationships of the MSEs for Werner states on *q* (varying from 0 to 1) and different number of copies *N* using the cube measurement bases^9^. The fact that the MSE of PLRE is larger than that of LRE demonstrates that the process of pulling 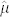 back to a physical state further reduces the estimation error.

## Discussion

In the LRE method, data collection is achieved by performing measurements on quantum systems with given measurement bases. This process can also be accomplished by considering the evolution of quantum systems with fewer measurement bases. For example, suppose only one observable *σ* is given, and the system evolves according to a unitary group {*U_t_*}. At a given time *t*, 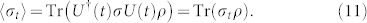
Suppose one measures the observable *σ* at time 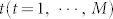 on *m* identically prepared copies of a quantum system. Denote the obtained outcomes as 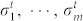, and their algebraic average as 
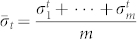
. Note that 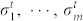 are independent and identically distributed. According to the central limit theorem^26^, 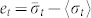 converges in distribution to a normal distribution with mean 0 and variance 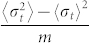. We have the following linear regression equations 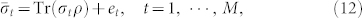
which are similar to (4). Hence, we can use the proposed LRE method to accomplish quantum state tomography.

The LRE method can also be extended to reconstruct quantum states with a prior information^12^,^27^,^28^,^29^ or states of open quantum systems. Actually, LRE can be applied whenever there are measurable quantities that are linearly related to all density matrix elements of the quantum system under consideration.

In conclusion, an efficient state reconstruction algorithm of linear regression estimation has been presented for quantum state tomography. The computational complexity of LRE is *O*(*d*^4^), which is much lower than that of MLE and BME. We have analytically provided an MSE upper bound for all possible states to be estimated, which explicitly depends upon the used measurement bases. This analytical upper bound can assist to identify optimal measurement sets. The LRE method has potential for wide applications in real experiments.

## Methods

### The recursive LS algorithm

For 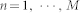, define 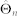 as 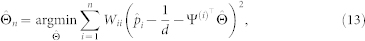
where *W_ii_* is the *i*-th element of the diagonal of *W*, and 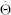 is an estimate of Θ. Hence, the LS solution 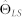 is equal to 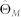. From (7), we have 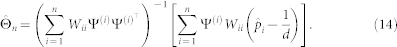
Define 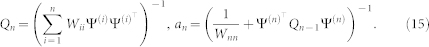
Using the matrix inversion formula (see, e.g., page 19 of^30^) 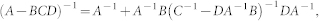
we have 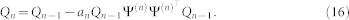
From (14), (15) and (16), the recursive form of 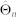 can be obtained as 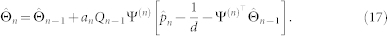
Note that *Q_n_* is not always invertible, especially when *n* is small. In order to apply the recursive algorithm in this case, one may choose the initial value in (16) *Q*_0_ being a given positive matrix, while 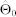 being a given vector. From (16) and the matrix inverse formula, one has 
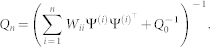


Hence, the recursive LS algorithm can still be applied. Although the solution obtained from (17) may be slightly different from the solution obtained using (14), this does not affect the asymptotic properties of the LS solution.

### The minimum of the MSE upper bound

The MSE upper bound of 2-qubit states is 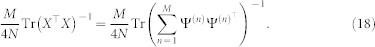
Minimizing this MSE upper bound is equivalent to minimizing Tr(*X*^T^*X*)^−1^.

Denote the eigenvalues of *X*^T^*X* as 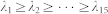. Since for all possible measurement bases, we have 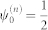, 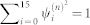 for 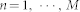, the problem is converted into the following conditional extremum problem: 
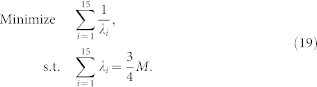
It can be proven that 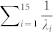 reaches its minimum 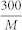 when 
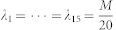
. Hence, the minimum of the MSE upper bound 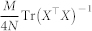 for all possible measurement bases is 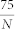 It can be verified that this minimum MSE upper bound can be reached by using the mutually unbiased measurement bases.

If only local measurements can be performed, i.e., 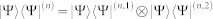, 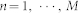, where |Ψ〉〈Ψ|^(*n*,1)^ and |Ψ〉〈Ψ|^(*n*,2)^ can be parameterized as 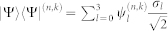, *k* = 1, 2. And we have 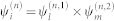, where *i* = 4*l* + *m*.

Due to additional constraints 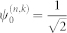, 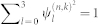, for *k* = 1, 2, and 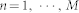, the problem of minimizing the MSE upper bound can be converted into the following problem: 
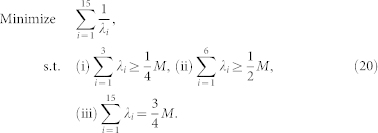
It can be proven that 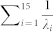 reaches its minimum 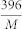 when 
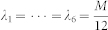
, 
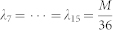
. Hence, the minimum of the MSE upper bound 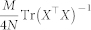 is 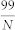. This minimum MSE upper bound can be reached by using the 2-qubit cube or tetrahedron measurement set.

## Author Contributions

B.Q., L.L. and D.D. developed the scheme based on linear regression model, Z.-B.H., G.-Y.X. and G.-C.G. performed the numerical simulations. All authors discussed the results and contributed to the writing of the paper. G.-Y.X. supervise the project.
